# Dementia in the minds of characters and readers – A transdisciplinary study of fictional language

**DOI:** 10.1177/14713012251335067

**Published:** 2025-04-22

**Authors:** Paula Devine, Jane Lugea, Gemma M. Carney, Carolina Fernandez-Quintanilla, Jan Carson

**Affiliations:** School of Social Sciences, Education and Social Work, Queen’s University Belfast, UK; School of Arts, English and Languages, Queen’s University Belfast, UK; School of Social Sciences, Education and Social Work, Queen’s University Belfast, UK; Departamento de Filologías Inglesa y Alemana, 16741 University of Granada, Spain; Independent Researcher and writer

**Keywords:** personhood, linguistics, literary language, reading, fiction

## Abstract

The concept of personhood in dementia has maintained its status as the definitive approach to dementia care. Personhood works at both practical and philosophical levels to maintain the humanity of people with dementia. The project described in this article used the concept of personhood to design community-engaged research which harnessed the power of literary language to access the internal life of a person with dementia. Here we outline the design and methods in detail, homing in on our main conclusion that literary language is a powerful tool in helping diverse stakeholder groups access the person in dementia. The research comprised three inter-linked strands. In Strand One we built a corpus of dementia fiction from which we identified twelve extracts from contemporary novels offering the internal perspective of a person with dementia. Strand Two involved six weekly meetings of separate reading groups with four distinct stakeholder groups – student social workers, members of the public, family carers, and people with dementia. The four groups engaged in separate, facilitated discussions of the extracts. This aspect of the project is unique as to the best of our knowledge no previous research has analysed readers’ responses to extracts of fictional characters’ narration of living with dementia. Strand Three was led by a well-known writer and comprised a series of public events and outputs which engaged readers and authors of dementia fiction with the genre. A dementia fiction festival and writer workshops resulted in publication of an anthology of short stories which included stories addressing a deficiency of racial and ethnically diverse characters noted in our corpus. The article concludes by discussing how working across disciplines and sectors to engage with dementia as a cultural as well as a clinical challenge has the potential to facilitate the understanding and emphasis of personhood in dementia studies.

## Introduction

At both societal and personal levels, dementia is a challenging issue. As a result it has been subject to analysis by disciplines as diverse as social work, psychology, psychiatry as well as arts and humanities (Hughes, 2013; Logsdon, McCurry and Teri, 2007; McGovern, 2015; Swinnen, 2016). Dementia has been discussed and described as an illness, a condition, a disease and a phenomenon. It has been seen as a site of personal tragedy and an epidemic on society. The framing of the illness at both personal (Nilsson, 2022) and at societal level have been examined (Zeilig, 2014). Throughout, the idea of personhood in dementia (Kitwood, 1997) has maintained its credibility as the definitive approach to understanding dementia. Personhood works at both practical and philosophical levels to maintain the humanity of people with dementia through each stage of a progressive illness (McLean, 2007). Personhood applies to the humanity of carers and other stakeholders as it allows us to use all of our senses in working with people with dementia.

The project described here uses that concept of personhood to design a model of community-engaged research which harnesses the power of literary language to uncover the internal life of a person with dementia. This article outlines the project design and methods in detail, focusing in on our main conclusion that literary language is a powerful tool in helping diverse stakeholder groups access the person in dementia. And, more generally, the transdisciplinary approach adopted by the project (incorporating humanities and social sciences) can facilitate a more humane/human-centred understanding to this condition. The focus on methods will allow others to use and develop them in formal and informal settings. Detailed findings are reported elsewhere (Carney et al., 2023; Fernandez-Quintanilla et al., 2025; Lugea et al., 2025).

## Dementia and language

Dementia is overtly associated with loss – of language, of autonomy and of connection. Loss of language can mean the loss of communication, with many carers reporting that they feel sad at the loss of intimacy in long-term relationships (Large and Slinger, 2015, p. 173). Loss of language is central to the reporting of clinical symptoms of dementia - ‘in dementia, language becomes seemingly meaningless and is eventually lost’ (Hughes, 2013, p. 353). This loss of language is associated with loss of meaningful connection with others, the very centre of human personhood as ‘the life of human beings is characterised by language’ (*ibid*.). In practical terms, the loss of language that people with dementia (and Alzheimer’s Disease in particular) experience has been itemised – ‘lack of cohesive discourse, irrelevant or tangential content, word-finding difficulties, and the use of semantically empty words such as pronouns (he, she, it) where the referent cannot be unambiguously identified’ (Mok and Müller, 2014, p. 835). It was with these losses and clinical deficiencies in mind that we devised a project which uses literary language to bring to life the internal subjectivity and experience of characters with dementia, giving readers access to their rich inner life, even if they no longer express themselves through ‘speech'.

## Aim

The purpose of this study was to use the internal narration of fictional characters with dementia to explore how stakeholders – student social workers, members of the public, family carers, and people with dementia – respond to narratives of living with dementia including depictions of loss of language and connection.

## Methods

**Figure 1.**
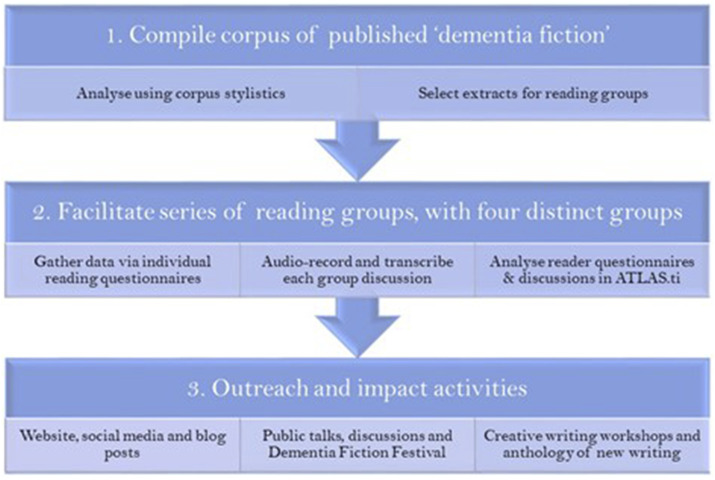
Project outline.

Thus, the project set out to investigate whether fictional language can be used to explore the lived experience of dementia. It ran from 2020 to 2022, with ongoing dissemination and engagement activities up to and including 2024. The research comprised three interlinked strands (see Figure 1).

## Design

### Strand One: Compile corpus of published ‘dementia fiction’

As a project with its intellectual roots in literary analysis, we drew upon the concept within Stylistics of ‘mind style’, which was first defined as ‘any distinctive linguistic presentation of an individual mental self’ (Fowler, 1977, p. 103). We used this method because it involved using creative literary language to depict the internal life of a fictional character with dementia. For instance, the following extract is narrated by one of the fictional characters with dementia used in our study:

‘A drip of something rolls down my forehead. It’s stifling in here. Boiling hot. I hoist myself off the sleeping-bench. There must be a cool-machine around here somewhere! Or a whirly-spinner that blows air around.’ (*The Things we Keep*, Sally Hepworth).

The use of the terms ‘sleeping-bench’ for *bed*, ‘cool-machine’ for *air-conditioning* and ‘whirly-spinner’ for *fan* indicate the anomic aphasia that can occur with dementia. It mirrors what is reported in clinical settings (Mok and Müller, 2014). Fictional representations can provide us with a useful mechanism by which dementia can be represented as a heterogenous condition, highlight how it is experienced by the individual person, and understand how dementia is understood by wider society.

In order to create the corpus, Lugea compiled a bibliography of contemporary fiction which focused on the internal perspectives of characters and narrators living with dementia. The texts needed to meet all the following six criteria: 1. Contemporary (i.e. published within the last 35 years); 2. Fictional (rather than autobiographical); 3. Prose; 4. Written in the English language; 5. Features a main character living with any type of dementia; 6. Presents the cognitive experience of a character living with dementia, either as a first-person account (with the person living with dementia as the narrator), or as a third-person account (with a separate narrator reporting on the characters, actions and events). In third-person accounts, narrators present the contents of characters’ minds either through Free Indirect Style (whereby the narrator’s report blends with the character’s consciousness) or psycho-narration (whereby the narrator can verbalise mental activity of which the character may not be conscious). The ability of a narrator to present the contents of a character’s mind is unique to fiction, given that, in real life, we cannot access other people’s minds in this way. For a description of these narrative strategies and how they can affect readers, see Cohn (1978) and Salem et al. (2017).

The corpus included twelve texts which met all six criteria, including two full short stories and ten novels, sometimes in part (see Table 1). While these texts vary in how they incorporate dementia, they all grant access to the internal perspective of the person living with dementia.

**Table 1.** Texts selected for dementia mind styles analysis.

Author	Publication year	Title	Character with dementia	Narration type	Number of words attributable to character with dementia
Smith, Ali	2011	*There But For The*	May Young (female, British)	Third-person, past tense	16,120
Dean, Debra	2006	*The Madonnas of Leningrad*	Marina Buriakov (female, Russian-American)	Third-person, present tense	7,591
LaPlante, Alice	2011	*Turn of Mind*	Jennifer White (female, American)	First-person, present tense	69,383
Harvey, Samantha	2009	*The Wilderness*	Jake Jameson (male, Jewish British)	Third-person, present tense	49,869
Healey, Emma	2015	*Elizabeth is Missing*	Maud Palmer (female, British)	First -person, present t tense	59,470
Hepworth, Sally	2016	*The Things we Keep*	Anna Forster (female, American)	Third-person, present tense	33,359
Genova, Lisa	2007	*Still Alice*	Alice Howland (female, American)	Third-person, past tense	70,317
Bernlef, Josef [translated by Adrienne Dixon]	1988	*Out of Mind*	Maarten Klein (male, Dutch-American)	First -person, present tense	48,720
Franzen, Jonathan	2010	*The Corrections*	Alfred Lambert (male, American)	Third-person, past tense	13,384
Munro, Alice	2012	'In sight of the lake' (short story)	Jean (female, Canadian)	Third-person, present tense	4,639
Rill, Eric	2015	*An Absent Mind*	Saul Reimer (male, Jewish Canadian)	First -person, present tense	14,401
Rogers, Jane	2012	'Hitting trees with sticks' (short story)	Celia Benson (female, British)	First -person, present tense	3,522
				Total	390,775

One of the texts - *Out of Mind* Bernlef (1988) - was originally written in Dutch. However, as outlined by Lugea (2022), the novel matched all six criteria. In particular, the stylistic techniques used by Bernlef, such as the change in narrator’s style across the novel and the reflection of the cognitive changes, were maintained within the English translation. The text was older than other texts, but nevertheless, fits within the timeframe of ‘contemporary’, as defined by Padley (2006).

Strand One involved both quantitative and qualitative data analysis, necessitating two slightly different data selection processes. The quantitative corpus analysis only included passages of narration attributable to the character with dementia, in order to be able to make claims about lexico-grammatical patterns in their narration. Once the data were selected, the texts were digitised by converting e-books to machine readable .txt files. Where this was not possible, paper copies of the books were scanned using Microsoft Lens. The resultant corpus consisted of 390,775 words (see Table 1). The quantitative analysis involved contrasting the narratives from this corpus of characters with dementia with a reference corpus of general fiction of over 14 million words (drawn from the British National Corpus), which allowed us to identify what is unique within our dataset. The texts were analysed using AntConc (Anthony, 2019).

For the qualitative analysis, the data selection process was less rigid as qualitative stylistic analysis involves considering the texts more holistically; for instance, including narratives from the perspectives of other characters allowed us to explore their influence and interaction. The data were analysed using *Nvivo*, using frameworks drawn from cognitive stylistics to identify features which contribute to the creation of dementia mind styles.

The analysis revealed the linguistic access points to the world-view of these fictional characters who were living with dementia, focusing on how the characters used linguistic devices such as metaphor, as well as on narrative structure and order, as clues to memory and perception. Resultant themes include memory and cognition in language and narrative, sensory and emotive experience, and the self and social relationships. The analysis highlighted the many ways that dementia can be experienced and represented, as well as the heterogeneity of the illness and the ways it can affect people (Lugea, 2022).

Strand One also involved selecting the extracts for the reading groups, based on the twelve texts used for the corpus. A full description of this selection process can be found later in this article.

### Strand Two: *Facilitate series of reading groups, with four distinct groups*

This strand comprised six sessions of four parallel reading groups which had the function of helping us understand if the stylistic effects we perceived impacted on real readers. We engaged in empirical reader response research within the tradition of empirical stylistics, which seeks to understand how readers react to texts (Hakemulder and van Peer, 2016; van Peer and Zyngier, 2008; Whiteley and Canning, 2017). This aspect of the project is particularly important as, to the best of our knowledge, no previous research exists that has elicited and analysed readers’ responses to dementia fiction. Over six weeks, the four groups engaged in separate, facilitated discussions of extracts of fiction depicting characters living with dementia.

#### Piloting the reading groups

Testing the methods was vital in ensuring the project’s success. Thus, a pilot reading group session was held in a community café in Belfast, Northern Ireland, involving participants with different relationships with dementia: an academic, a person living with dementia, practitioners from different organisations, and a writer. Two key lessons emerged. Firstly, the social and spatial context of the reading group sessions are important. For example, holding the reading group sessions in an informal location and providing refreshments (tea/coffee and cake) provides an opportunity for the participants to relax, interact and feel welcomed as ‘real readers’ in a naturalistic rather than experimental environment (Hall, 2009).

Secondly, the involvement of participants with differing experiences of dementia revealed potential problems. Several participants noted that they felt unconfident expressing their opinion among ‘experts’, such as people living with dementia, a writer, or practitioners. Some felt that not having lived experience of dementia gave them fewer rights and entitlement to talk about it. Therefore, the team decided that running four parallel and separate groups was more appropriate, each comprising participants with a similar relationship with dementia: A - student social workers from Queen’s University Belfast where the project was based; B - the general public; C - carers of someone living with dementia; and D - people living with dementia. This would give each group their own space to discuss their ideas, as well as reflecting the finding of Rosato et al. (2019) that life experience influences attitudes and understanding. Student social workers were included as they are in the process of developing their professional stance and were likely to encounter people with dementia in their future careers.

#### Recruitment of participants

Readers were purposively sampled for each of four reading groups. Group A were a convenience sample, as the team had easy access to them via the university, and Group B were recruited via social media. Group C were recruited via Alzheimer’s Society, whilst Group D were recruited via DementiaNI (a local empowerment charity for people living with dementia).

#### Devising the reading groups

The reading groups were due to take place in person in locations across Northern Ireland. However, due to the COVID-19 pandemic, all reading groups were held virtually, resulting in technical, research and ethical issues.

Initially, the research team perceived this move to online as a retrograde step, especially given that the pilot stage highlighted the importance of ‘tea and buns’ and social interaction. Nevertheless, there were several advantages to an online format. It enabled geographical spread of participants, and thus more diversity, a benefit previously highlighted by Matthews et al. (2018). In particular, Group C (carers) participants lived across the United Kingdom (UK), rather than in one locality. Participants did not have to spend time travelling to the venue, which was particularly beneficial for carers, many of whom, due to their caring responsibilities, would not have been able to devote additional travel time.

Group A (student social workers) met via Microsoft Teams, as this was the platform used within their university course. Groups B, C and D were facilitated using Zoom, as by 2021, this was the platform with which most users were familiar. The project team developed comprehensive guidelines on facilitating online workshops which drew upon previous research (Archibald et al., 2019; Davies et al., 2020; Matthews et al., 2018; MacNamara et al., 2021; Tuttas, 2015).

Particular attention was paid to developing guidelines for people living with dementia. Working within the parameters of ‘inclusive research’ (Nind, 2017), the research team met with members of DementiaNI (all of whom are living with dementia). As had become the norm by this time, all DementiaNI group meetings were taking place online, and so the members reassured the project team that they were comfortable using Zoom. In order to further facilitate accessibility and familiarity, the project team used the same Zoom meeting room that DementiaNI members used for their regular support meetings. DementiaNI members highlighted practical issues relating to the reading groups, such as an optimal number of group participants (4–6), and the need for larger font size in printed material. Some members no longer could read long passages as they found it increasingly difficult to retain information, and so recommended that the length of extracts for the reading group should be limited. Several members said that they missed reading and had made the transition to audio books. Therefore, playing an audio recording of the extracts would enable full participation of everyone, regardless of their level of engagement with the written text. These comments and suggestions reinforced messages from the guidelines produced by the Dementia Engagement and Empowerment Project (DEEP) when collecting the views of people living with dementia (DEEP, 2013). Based on these discussions, as well as the DEEP recommendations on writing dementia-friendly information (DEEP, 2013b), Group D participants explored one extract in each reading group session. Other reading groups read the same extract, plus a second, longer extract each session (see Table 2).

**Table 2.** Reading group extracts.

Session	Extract 1 (all groups)	Extract 2 (groups A, B and C)
1	*An Absent Mind *(Eric Hill)	*Elizabeth is Missing* (Emma Healey)
2	*The Madonnas of Leningrad* (Debra Dean)	*The Madonnas of Leningrad*
3	*The Wilderness* (Samantha Harvey)	*The Wilderness*
4	*Still Alice* (Lisa Genova)	*Turn of Mind* (Alice LaPlante)
5	*Out of Mind* (J. Bernlef)	*Out of Mind*
6	*The Things we Keep* (Sally Hepworth)	*There But For The* (Ali Smith)

#### Ethics

Ethical approval was obtained from the relevant ethics committee within Queen’s University Belfast. Each potential participant was emailed an information sheet giving details about the project, a reminder that participants were free to withdraw from the project at any time, and confirmation that all their information would be treated confidentially. They then completed the online consent form.

Participants were asked to fill in an online questionnaire focusing on their demographic and socio-economic background (including age, sex, occupation and education). The questionnaire explored the participants’ experience and frequency of reading, along with the type of books that they enjoyed. The final set of questions asked about knowledge of, and relationship to, someone living with dementia, although these were omitted for Group D participants.

Those in Groups A, B or C completed a second questionnaire, exploring their levels of empathy, and their understanding of dementia. The questionnaire included the Interpersonal Reactivity Index (IRI) (Davis, 1983), which is used to measure people’s trait or dispositional empathy. Examples of the 28 items include ‘I really get involved with the feelings of the characters in a novel’ and ‘Other people’s misfortunes do not usually disturb me a great deal’.

Once respondents had returned their completed consent form, they were sent a pack containing a hand-drawn thank you card, and an envelope for each of the six reading group sessions. Each envelope contained a printed copy of the relevant extracts and a stamped-addressed envelope, as well as a hot drink sachet and a small packet of biscuits. This reflected the importance of refreshments in helping to bond the group as highlighted during the pilot session. The pack sent to participants in Groups A, B and C also contained a leaflet which provided information about dementia, and links to sources of support for people living with dementia and for carers. Group D participants were also sent a paper questionnaire exploring responses to each extract (Groups A, B and C completed this online).

#### Selecting the extracts

Following on from the quantitative analysis in Strand One, Lugea identified 28 extracts from twelve novels or short stories. That analysis confirmed that these extracts reflected a wide representation of dementia and characters. The extracts were reviewed by the research team and whittled down to twelve extracts (Table 2), each comprising 500–1,000 words. These reflected a diversity of character types (such as male/female), experiences in the character with dementia (e.g., memory loss/failure/flashback, humour, sensory awareness, emotions, interactions with others), stage of dementia, stylistic features (such as metaphor, lexico-grammatical patterns such as marked transitivity and underlexicalisation, repetition, given/new information), and narrative styles (first person, third person, present tense, past tense). The team was careful to ensure that the extracts for Group D did not incorporate scenarios which may be emotionally distressing.

#### Reading groups

The reading group sessions took place in April and May 2021. For Groups A, B and C, the sessions lasted 90 minutes, and the groups engaged with two extracts. Sessions for Group D ran for 60 minutes, focusing on one extract. Sessions were facilitated by one or two members of the research team, and started with a welcome and general discussion. The sachets of refreshments provided a useful talking point to break the ice. This was followed by an (re)introduction to the research project and the running order of the session.

The extracts were used to generate verbal discussion, as well as textual and questionnaire data. Moreover, the second function of the Reading Groups was to assess the effect of the fictional dementia mind styles on readers, in terms of emotional and empathetic engagement. For each extract, the process was the same. Firstly, the facilitator provided a short introduction and context to the extract. An audio recording of the extract was played, and participants could read along with the paper copy of the text. Participants were asked to make notes on this paper copy, highlighting any words, phrases or sentences that they particularly engaged with, either positively or negatively. Following this stage, participants in Groups A, B and C completed an online questionnaire exploring their engagement with the extract and the characters, made up of closed and open questions, short and long answers as well as quantitative and qualitative responses (see Appendix 1, Supplemental Material). Group D completed a paper version, which was shorter, questionnaire (see Appendix 2, Supplemental Material). As well as being shorter, the questions focused on how the fictional representation tallied with their experience, rather than how they identified with the characters.

The format of the questions for Groups A, B and C was the same for all extracts. Questions included:

 • Did you feel like the extract was presented through a particular character’s eyes?
 • To what extent could you understand the character with dementia’s point of view?
 • What was it about the extract that made you understand his/her viewpoint? (only asked if the participant indicated that they could understand this character’s point of view to some degree)
 • To what extent could you understand her carer’s point of view?
 • Can you describe the emotion(s) you felt while reading?
 • How do you think the character with dementia was feeling?
 • Did reading this extract make you think about the experience of living with dementia in a new or different way?
 • Was there anything in this extract that contradicts your experience of dementia?

After completing the questionnaire, all groups engaged in a 15–20-minute discussion about their responses to the extract and the characters. Potential limitations of the reading group method include social desirability bias and self-censorship (Lang, 2009, p. 325). Therefore, in order to mitigate these effects, the questions asked during the discussion were open-ended, in order to minimise researcher control (Whiteley and Canning, 2017), and the facilitators avoided giving their own opinion in order not to bias participants towards saying what they believe the researcher wants them say (‘demand effects’).

#### After the reading groups

After the six reading group sessions had taken place, participants were asked to return their marked-up extracts in a stamped-addressed envelope that had been included in their initial pack. Those in Groups A, B or C were asked to complete an online questionnaire. As well as repeating questions relating to the Interpersonal Reactivity Index, this questionnaire explored the impact of the reading groups, including if and what new things participants had learned about dementia, and if and how the reading groups impacted their awareness and understanding of dementia, their behaviour in situations involving dementia, and their reading practices. Group D members did not complete these final questionnaires. Instead, at the end of the sixth session, there was a discussion exploring reactions to, and evaluations of the reading groups.

As a thank you for participating in the sessions, those participants who had participated in at least five sessions were sent a £20 book token. Participants in Group D were given a £20 shopping voucher, as some members had noted that their condition meant that although they no longer read books, they now enjoyed audio books.

Throughout Strand Two, the team collected a wide array of data. The qualitative data collected via audio recordings of the reading group discussions, and marked-up questionnaires were analysed using *Atlas.ti*. The quantitative survey data were analysed via *SPSS* (version 29), and were also input into *Atlas.ti* for triangulation with the qualitative data.

#### Participation

A total of 31 people were recruited for the reading groups. Tuttas (2015) and Matthews et al. (2018) report high rates of attrition when facilitating online discussion/focus groups. However, in this project there was excellent take up and retention over the six weekly sessions, with 25 participants attending at least five of the six sessions. Full details of participants including demographics, socio-economic status and educational qualifications are available in Appendix 3, Supplemental Material. All but one participant was white, with English as their first language. This reflects the relative homogeneity of the local population (Carney et al., 2023).

### Strand Three – outreach and impact activities

Strand Three used the findings from Strands One and Two to improve societal awareness and understanding of dementia, and to maximise the impact of the project and its findings. These diverse activities included creative writing workshops and a Dementia Fiction Festival in September 2021, as well as academic papers, blogposts, international conference papers in linguistics and gerontology and invited public talks. The project commissioned an anthology of short stories inspired by dementia (Carson and Lugea, 2022). Three of these activities will be discussed in more detail below, in order to give a flavour of the range and scale of these events.

#### Online conversations with authors of dementia fiction

Facilitated by Carson, the project’s impact and outreach officer, this online conversation featured two authors (Wendy Mitchell and Anna Wharton) discussing their experiences and reflections on writing about dementia. This event took place in February 2021 as part of the Northern Ireland Science Festival.

#### Dementia Fiction Festival

The two-day Dementia Fiction Festival was held online in September 2021. As well as performances of new writing, the festival featured workshops, panel discussions and keynote talks from researchers, creative writers, and people living and working with dementia. The topics included the ethics of writing about dementia, exploring issues such as dignity, research and appropriation; diversity in dementia narratives, highlighting the lack of diverse representations, and ways in which we can foster more diversity in writing and thinking about dementia; portrayals of dementia in fictional and personal accounts, and how these can undermine or support, educate or misinform; writing fiction, memoir and biography relating to dementia; dementia narratives in children’s and young adult literature; and innovative arts projects which engage people living with dementia, their friends, family and carers in reading, writing and theatre. One key session comprised a one-to-one chat with a member of DementiaNI, focusing on what it is like to live with dementia, and how writers should approach writing about the condition.

Over 200 people participated throughout the festival. While it was disappointing not being able to meet face-to-face, one advantage of a virtual event was that participants joined from all over the world, including across the UK and Ireland, Switzerland, Germany, Spain, Norway, India, and Aotearoa New Zealand.

29 participants completed an evaluation form at the end of the festival. Responses were rich and varied, particularly around the value of listening to people living with dementia and how their attitudes and expectations of people with dementia shifted after taking part in the festival. Most participants strongly agreed that they learned something about living with dementia; that it was important to highlight some of the ethical issues when writing about dementia, and that reading about characters with dementia can help our understanding of living with the condition. The following comments illustrate these issues:

• *Hearing directly from people living with dementia about what they feel about fictionalisations of their experiences (authenticity, humour etc) was enlightening.*
• *I assumed that quality of life was so poor for people with dementia that it was barely worth living. My attitude was patronising and could have been detrimental to those with the condition so I am grateful to have been challenged on this.*
  In terms of challenges, the lack of racial diversity of authors and characters within dementia fiction was identified as an issue.

#### Anthology of short stories

An anthology of short stories was commissioned as part of the project (Carson and Lugea, 2022), with each of the 14 stories disrupting perceived notions of what dementia is, with the aim of breaking down the stigma experienced by those living with the condition. The title of the collection – *A Little Unsteadily into Light* - was inspired by a stage direction in Samuel Beckett’s *Krapp’s Last Tape.* While that play is not about dementia, it explores relevant themes of memory, ageing and identity.

The anthology included pieces from eminent authors (such as Sinéad Gleeson, Caleb Klaces and Nuala O’Connor, one of whom won an award with the story they had written for the collection), many of whom were motivated to write about dementia based on their own experience of family or friends with the condition. However, in order to provide an opportunity to emerging authors, two stories were written by students of creative writing workshops facilitated by Carson and Lugea. The workshops focused on how to write well and ethically about dementia and were open to 12 fiction writers based in the UK or Ireland. Participants submitted their idea for a short story, of which two were selected. These authors received mentorship and advice from Carson, and their stories were included in the anthology. The royalties from the book go to DementiaNI. Launch events in two cities gave an opportunity to engage the general public in the project’s themes.

Most fictional characters explored in Strands One and Two of the project were female, old, white, or middle-class. We sought to address this lack of diversity within the anthology, which includes one by a Syrian-Irish author, another by a British-Ghanaian author and several exploring LGBTQ + experiences of dementia.

## Limitations of the study

One limitation of the criteria we used to select dementia fiction is that the focus on texts in English means that the findings are only generalisable to anglophone cultural representations. Another limitation was that despite our wish to represent an ethnically diverse range of fictional characters with dementia in our study, most were white. One form of racial diversity that did appear in the novels we examined comprised paid carers from black/ethnic minority groups (Carney et al., 2023). Failure to address race and ethnic identity has been identified in dementia research more generally (Large and Slinger, 2015).

## Discussion and conclusion

This was a complex project which worked on several levels. It engaged with the literary language of dementia fiction academically, through the systematic creation of a corpus of dementia fiction. Secondly, we elicited and analysed the perspectives of readers and, finally, we engaged in a creative process whereby authors reflected on and produced new dementia fiction. The full cycle of literary life was therefore included in the study and we believe that this resulted in a rich set of research results that are applicable more broadly.

In Strand One literary concepts such as under-lexicalisation (loss of specific words) and aphasia as well as more familiar concepts such as metaphor were operationalised in support of better understanding the person in dementia. It may seem paradoxical to use complex literary language and concepts to understand the experience of living with a condition which erases language. However, we found the level of linguistic precision and creativity displayed by authors of the fictional extracts included in our study was key to producing credible first-person accounts of dementia. The responses elicited in the questionnaire, marked-up extracts and the discussion allowed us to explore ‘during-reading effects’ (Whiteley and Canning, 2017) in terms of how readers engage with the characters (e.g., interpretation, understanding, etc.). The significance of language was particularly apparent in the reported results of the reading groups (Carney et al., 2023; Fernandez-Quintanilla et al., 2025; Lugea et al., 2025) which demonstrated how the language used in the extracts spoke *to* and *for* the person in dementia. The analysis highlighted the many ways that dementia can be experienced and represented, as well as the heterogeneity of the illness and people affected.

Participants in our study had experienced dementia in an array of different ways. Some had an early-stage diagnosis, others had more advanced dementia. Some readers in Group C (carers) had seen their partners lose their lives to dementia. Others had more limited experience. Likewise, the fictional characters in our study represented a range of stages and experience of dementia across different time periods and care settings.

We also engaged with authors of dementia fiction, in conversation, through the books we produced and as part of the dementia fiction festival. We conclude that by working across and between disciplines and sectors, and by engaging with two dementia charities as well as authors and readers, we found that literary language provides a basis for shared meaning of the full human experience i.e. living with dementia, whether as a carer or a person with a diagnosis. As language loss is such a central part of the clinical and cultural experience of living with dementia, finding ways to use literary language to speak *to* and *with* people with dementia is surely an important means of actioning the very essence of personhood.

In Strand Two, the findings from our reading groups support the work of Hutmacher and Schouwink (2022, p. 405) who found that ‘engaging in creative activities can have an impact on an experiential, cognitive and emotional, as well as on a behavioural and interactional level.’ The people living with dementia in our study found reading about their condition, particularly in a group setting, to be enjoyable and empowering (see Carney et al., 2023). For these reasons, we have reported the detail of our methods here so that dementia practitioners can use them in informal or residential care settings. People living with dementia reported a high level of satisfaction with the process of facilitated reading in a group. They requested to be allowed to continue and now Lugea is leading a new project *Still Reading* which works with The Reader and DementiaNI to train dementia facilitators in our methods. However, rather than focusing on eliciting the responses of readers to dementia fiction, the new project focuses on reading literature in general (prose and poetry) in group settings with people living with dementia. While arts-based interventions are using increasingly, these are usually music or craft-based, with literary interventions often overlooked. This is despite evidence for the positive impact of ‘shared reading’ on the mental health, mood, confidence, and social inclusion of people with dementia (Clark et al., 2019; DeVries et al., 2019; Orrell et al., 2019). Building on the strong relationship forged with DementiaNI through the research reported here, Lugea is co-designing a method of shared reading with people living with dementia with accessibility and empowerment at its core.

Finally, we return to our shared aim to find ways to enact and operationalise the person in dementia. It is well established that it is challenging to provide verifiable evidence of how the arts enrich the lives of people living with dementia. Focusing on language and dementia is a powerful way of examining, not only what dementia represents in terms of loss, but also how connection can be maintained. Establishing dementia as a viable human experience, particularly in deep old age, is central to operationalising Kitwood’s ideal of personhood. We need language, often in its most sophisticated and creative forms, to do this (see Swinnen, 2016). Moreover, we believe that adopting multi-strand and transdisciplinary approaches which engage a range of stakeholders on cultural as well as clinical levels, is the most effective way that the person in dementia can be found.

## Supplemental Material

Supplemental Material - Dementia in the minds of characters and readers – A transdisciplinary study of fictional language Supplemental Material for Dementia in the minds of characters and readers – A transdisciplinary study of fictional language by Paula Devine, Jane Lugea, Gemma Carney, Carolina Fernández-Quintanilla, and Jan Carson in Dementia
