# Supplementary material for: Dementia in the minds of characters and readers – A transdisciplinary study of fictional language
Source: Dementia (London). 2025 Apr 22;25(2):350–65. doi: 10.1177/14713012251335067 (PMC12816396; doi:10.1177/14713012251335067)
Supplement: Supplemental Material - Dementia in the minds of characters and readers – A transdisciplinary study of fictional language [file sj-pdf-1-dem-10.1177_14713012251335067.pdf]

Supplementary material

Appendix 1. Example Reading Questionnaire for Groups A, B and C, Extract 1a

Please complete this questionnaire after reading - and marking-up - extract 1a from *An Absent Mind*.

1. Looking at the markings you made on the extract, how and why did those bits strike you?

____

2. Have you read this book, *An Absent Mind*, before?

□ Yes

□ No

3. Do you have personal experience of anything in this extract?

□ Yes

□ No

4. If yes, can you explain?

____

5. Did you feel like the extract was presented through a particular character's eyes?

□ Yes

□ No

6. If yes, whose eyes?

□ Saul

□ Monique

□ Other

7. If you selected 'other', whose eyes do you think the extract is presented through?

____

8. To what extent could you understand Saul's point of view?

□ Not at all

□ Slightly

□ Moderately

□ Considerably

□ Completely

If response is ‘Not at all’, skip to Q10

9. What was it about the extract that made you understand Saul’s viewpoint?

____

10. To what extent could you understand Monique’s point of view?

□ Not at all

□ Slightly

□ Moderately

□ Considerably

□ Completely

If response is ‘Not at all’, skip to Q12

11. What was it about the extract that made you understand Monique’s viewpoint?

____

12. How strong were the emotion(s) you felt during reading?

□ None

□ Mild

□ Moderate

□ Significant

□ Very strong

13. Can you describe the emotion(s) you felt while reading? If your emotion(s) relate to a specific moment in the extract, please specify.

____

14. If the emotions you felt relate to a specific character, please specify:

____

15. How do you think Saul, the character with dementia, was feeling?

____

16. To what degree did you feel the same emotions as Saul?

□ Not at all

□ Slightly

□ Moderately

□ Considerably

□ Completely

17. If you felt a different emotion, can you name it:

____

18. Did reading this extract make you think about the experience of living with dementia in a new or
different way?

□ Yes

□ No

19. If you answered *yes*, please explain:

____

20. Was there anything in this extract that contradicts your experience of dementia?

□ Yes

□ No

Not applicable

21. If you answered *yes*, please explain:

____

22. Any other comments on this extract? You can use this space to provide responses that you may not want to give in the group discussion. ______________________

23. Please enter your first name and surname:

____

24. Please select the reading group you are participating in:

□ A. Social Work students

□ B. General Public

□ C. Carers

Appendix 2. Example Reading Questionnaire for Group D, Extract 1a

This questionnaire is to be completed during our reading group meeting. We will do it after reading the extract from ‘An Absent Mind’.

1. If you made markings on the extract, how and why did those bits strike you?

---

2. This extract is from the book ‘An Absent Mind’. Have you read this book before?

□ Yes

□ No

3. Do you have personal experience of anything in this extract?

□ Yes

□No

4. If yes, can you explain?

---

5. Did reading this extract make you think about the experience of living with dementia in a new or different way?

□ Yes

□ No

6. If you answered yes, please explain:

---

7. Was there anything in this extract that contradicts your experience of dementia?

□ Yes

□ No

8. If you answered yes, please explain:

---

9. Any other comments on this extract? You can use this space to provide responses that you may not want to give in the group discussion.

---

10. Please enter your full name:

____

Appendix 3: Demographic and socio-economic characteristics of participants

**Group**	A: Student social workers (9)	B: General public (9)	C: Carers (6)	D: People living with dementia (7)
**Gender**	6 female, 3 male	8 female, 1 male	4 female, 2 male	4 female, 3 male
**Age**	7 aged 18-29, 2 aged 39-49	18-69	18-69	40-79
**Attendance**	7 attended at least 5 sessions	8 attended at least 5 sessions	6 attended at least 5 sessions	4 attended at least 5 sessions
**Occupation**	All student social workers	Psycho-therapist, civil servant, shop owner, publishing, retail, teacher	Hospitality, engineering, self-employed, human resources, nursing	Building, hairdressing, driving, disability support worker
**Educational attainment**	All in higher education, all had A level, 2 had undergraduate degree, 1 had postgraduate	All had A level or higher, 4 had undergraduate degree, 3 had postgraduate degree	2 had CSE, 3 had A level, 1 had undergraduate degree	3 had no qualifications, 1 had GCSE, 2 had undergraduate degree.

	degree			
